# Draft Genome Sequence of *Enterococcus mundtii* QAUEM2808, Isolated from Dahi, a Fermented Milk Product

**DOI:** 10.1128/genomeA.00995-16

**Published:** 2016-09-15

**Authors:** N. Farah, A. Mehdi, S. I. Soomro, N. I. Soomro, R. Tareb, N. Desmasures, J. P. Vernoux, S. M. Bakhtiar, M. Imran

**Affiliations:** a Department of Microbiology, Faculty of Biological Sciences, Quaid-i-Azam University, Islamabad, Pakistan; b Department of Bioinformatics and Biosciences, Capital University of Science and Technology, Islamabad, Pakistan; c Normandie Université, UNICAEN, UNIROUEN, ABTE, Caen, France

## Abstract

*Enterococcus mundtii* QAUEM2808 has been isolated from dahi, an indigenous fermented milk product of Pakistan. Here, we report the draft genome sequence for this strain, which consists of 160 contigs corresponding to 2,957,514 bp and a G+C content of 38.5%.

## GENOME ANNOUNCEMENT

*Enterococcus mundtii* is a Gram-positive, facultative, anaerobic, yellow-pigmented, nonmotile bacterium typically isolated form soil, plant surfaces, cows’ teats, and milkers’ hands (1). This bacterium does not have catalase and cytochrome oxidase enzymes. It can ferment carbohydrates via homolactic glucose metabolism (2) and has a low G+C content ranging between 38 and 39% (3). It is rarely associated with human infection (4). Some strains have been proposed to be used as probiotics to prevent mastitis in cows (5). Coculture of *E. mundtii* and *Lactobacillus plantarum* prevented the growth of *Listeria monocytogenes* ScottA under a simulated gastrointestinal model with infant milk formulations as substrates (6, 7). Here, we present the draft genome sequence of a unique strain, *E. mundtii* QAUEM2808, which was isolated from the fermented milk product dahi.

The genome sequencing of QAUEM2808 was performed using a HiSeq 2000 system (Macrogen, South Korea) to generate Illumina paired-end reads with a coverage of >100×. The resulted HiSeq reads were assembled using Velvet software (8). The assembly gave rise to a draft genome that consists of 160 contigs corresponding to 2,957,514 bp and a G+C content of 38.5%. These data are comparable to genomes that have already been reported (9). Genome annotation of QAUEM2808 was performed using the RAST server (10) and the NCBI Prokaryotic Genome Annotation Pipeline. The genome was predicted to contain a total of 2,701 genes, having 2,586 coding sequences, 24 RNAs, and 91 pseudogenes.

A preliminary analysis of this draft genome enabled us to identify a bile salt hydrolase belonging to the choloylglycine hydrolase family of enzymes, which indicates the potential ability to survive in the gastrointestinal tract (11). Although some strains of *E. mundtii* can produce bacteriocin, the genome of the present strain did not have genes related to known bacteriocin production. The presence of a high number of pseudogenes indicates the adaptation of this strain in microbial communities and in the nutritional-rich dairy matrix, as reported for dairy lactic acid bacteria (12). Regarding biogenic amines, two copies of each tyrosine and lysine decarboxylase were identified, but the histidine decarboxylase gene was not found. Genomic islands and virulence/resistance gene annotation were analyzed through VFDB (13) and ARDB (14). It was found that most significant virulence factors frequently associated with clinical isolates of enterococci are not present in our genome, as reported earlier for *E. mundtii* (15). A more comprehensive safety assessment for this strain will be reported in a future publication.

Draft genome sequences of some *E. mundtii* originating from both dairy and nondairy sources have been published in recent years (9, 16, 17).

### Accession number(s).

This whole-genome shotgun project has been deposited at DDBJ/ENA/GenBank under the accession number LSMC00000000. The version described in this paper is the first version, LSMC0100000.
